# Inhibition of NADPH oxidase 1 activity and blocking the binding of cytosolic and membrane-bound proteins by honokiol inhibit migratory potential of melanoma cells

**DOI:** 10.18632/oncotarget.6860

**Published:** 2016-01-09

**Authors:** Ram Prasad, John C. Kappes, Santosh K. Katiyar

**Affiliations:** ^1^ Department of Dermatology, University of Alabama at Birmingham, Birmingham, AL, USA; ^2^ Departments of Medicine and Pathology, University of Alabama at Birmingham, Birmingham, AL, USA; ^3^ Environmental Health Sciences, University of Alabama at Birmingham, Birmingham, AL, USA; ^4^ Nutrition Obesity Research Center, University of Alabama at Birmingham, Birmingham, AL, USA; ^5^ Comprehensive Cancer Center, University of Alabama at Birmingham, Birmingham, AL, USA; ^6^ Birmingham Veterans Affairs Medical Center, Birmingham, AL, USA

**Keywords:** honokiol, melanoma, NADPH oxidase, cell migration, imaging

## Abstract

Overexpression of NADPH oxidase 1 (Nox1) in melanoma cells is often associated with increased migration/metastasis rate. To develop effective treatment options, we have examined the effect of honokiol, a phytochemical from *Magnolia* plant, on the migratory potential of human melanoma cell lines (A375, Hs294t, SK-Mel119 and SK-Mel28) and assessed whether Nox1 is the target. Using an *in vitro* cell migration assay, we observed that treatment of different melanoma cell lines with honokiol for 24 h resulted in a dose-dependent inhibition of cell migration that was associated with reduction in Nox1 expression and reduced levels of oxidative stress. Treatment of cells with N-acetyl-L-cysteine, an anti-oxidant, also inhibited the migration of melanoma cells. Treatment of cells with diphenyleneiodonium chloride, an inhibitor of Nox1, significantly decreased the migration ability of Hs294t and SK-Mel28 cells. Further, we examined the effect of honokiol on the levels of core proteins (p22*^phox^* and p47*^phox^*) of the NADPH oxidase complex. Treatment of Hs294t and SK-Mel28 cells with honokiol resulted in accumulation of the cytosolic p47^phox^ protein and decreased levels of the membrane-bound p22^phox^ protein, thus blocking their interaction and inhibiting Nox1 activation. Our *in vivo* bioluminescence imaging data indicate that oral administration of honokiol inhibited the migration/extravasation and growth of intravenously injected melanoma cells in internal body organs, such as liver, lung and kidney in nude mice, and that this was associated with an inhibitory effect on Nox1 activity in these internal organs/tissues.

## INTRODUCTION

Melanoma remains the leading cause of skin cancer-related deaths due to its propensity to metastasize at distant body organs. The average survival of human patients with advanced stage melanoma is less than one year because of lack of effective therapies once the melanoma has spread to vital organs [1, 2]. Although melanoma is less common than other type of cutaneous malignancies, it causes approximately 75% of skin cancer-related deaths [1, 2]. Extensive efforts have been made to understand the causes and mechanisms of melanoma progression, but the control of melanoma metastasis remains a significant clinical challenge [2, 3]. The American Cancer Society indicated that the incidence of melanoma is increasing, particularly in children [2, 4]. Therefore, an approach that reduces the risk of melanoma metastasis or invasion may facilitate the development of an effective strategy necessary to improve outcomes in patients suffering from melanoma.

NADPH oxidase (Nox) is a family of enzymes that catalyzes transfer of an electron from NAD(P)H to an oxygen molecule to generate superoxide or hydrogen peroxide. Nox1, a catalytic subunit of NADPH oxidase, is overexpressed in melanoma [5]. Nox1 plays an important role in reactive oxygen species (ROS) production, and overproduction of intracellular ROS has been considered as a risk factor in cancer development. Increased generation of Nox1-derived ROS is functionally required for Ras transformation phenotypes [6], upregulation of vascular endothelial growth factor (VEGF), tumor progression and tumor cell migration [6–8]. Ras-transformed cells are highly metastatic, and the Ras oncogene is able to stimulate both matrix metalloproteases (MMPs) production and cell migration [9].

Bioactive phytochemicals that are non-toxic at effective doses have been tested against multiple tumor models. These small molecule phytochemicals offer promising options for the development of effective chemotherapeutic or chemopreventive agents. Honokiol, a small molecular weight molecule extracted from the *Magnolia* plant species, has been reported to have anti-cancer properties in various animal tumor models, such as, non-melanoma skin cancer, breast, lung and prostate cancers [10–15] with no apparent signs of toxicities in these models. However, the anti-metastatic potential of honokiol against melanoma is largely unexplored. In this study, we examined the effect of honokiol on the migration potential of melanoma cancer cells, as the migration or invasion of cancer cells is a major event in the metastatic cascade of cancers. For this purpose, we used various human melanoma cancer cell lines as an *in vitro* model and verified our findings using *in vivo* athymic nude mice as a tumor cell invasion model. Furthermore, we ascertained that the inhibitory effect of honokiol on melanoma cell migration is mediated through the inhibition of Nox-1 and associated molecular targets.

## RESULTS

### Basal level of Nox1 protein in different melanoma cancer cell lines

**Figure 1:**
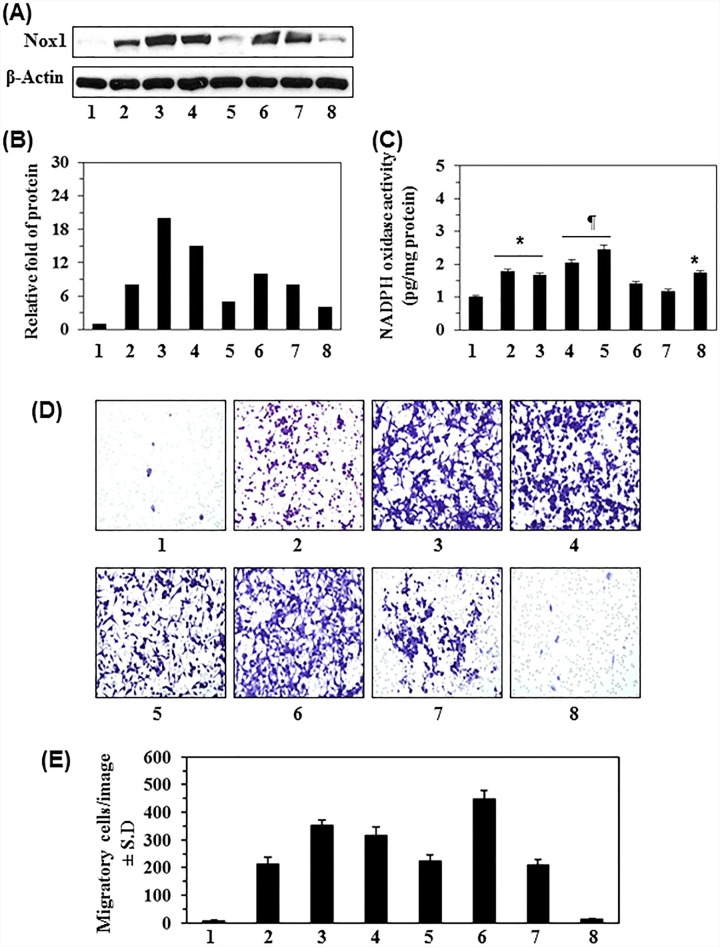
Basal level of NADPH oxidase 1 (Nox1) in normal human melanocytes (NHM) and various human melanoma cell lines and their migration ability **A.** Relative basal levels of Nox1 protein expression in normal human melanocytes and human melanoma cell lines were analyzed using western blot analysis. **B.** Relative levels of Nox1 protein in cells determined by the analysis of band intensity as detailed in Materials and Methods. **C.** Nox activity was measured using a colorimetric assay in NHM and melanoma cells, and has been expressed as pg/mg protein. Significantly higher in melanoma cells *versus* NHM; *P<0.05, ^¶^*P*<0.01. **D.** Migration capacity of melanoma cells in comparison to NHM. An equal number of human melanoma cells and NHM were analyzed for cell migration using Boyden chambers. After 24 h, migratory cells were detected on the membrane after staining the migratory cells with the 0.1% crystal violet dye. Representative photomicrographs are shown. **E.** The migratory cells were counted under microscope and the results are expressed as the mean number of migratory cells ± SD per image or field (*n* = 3). All melanoma cell lines and NHM are designated as: **1,** NHM; **2,** A375; **3,** Hs294t; **4,** SK-Mel 119; **5,** SK-Mel 28; **6,** Mel1241; **7,** Mel 928; and **8,** Mel 1011.

We first examined the basal level of Nox1 protein expression in different melanoma cell lines as compared with the levels in normal human melanocytes (NHM). As shown in Figure 1A, western blot analysis revealed that the melanoma cell lines (A375, Hs294, SK-Mel 119, SK-Mel 28, Mel1241, Mel1011, and Mel928) exhibited different basal levels of Nox1 expression. The basal level of Nox1 in NHM was detectable but to a lesser extent than observed in melanoma cell lines (Figure 1A). The densitometry analysis of bands indicated that the basal levels of Nox1 in melanoma cell lines were 4 to 20-fold higher than NHM (Figure 1B). Nox1 is one of several isoforms of NADPH complex; therefore, we further determined the total NADPH oxidase (Nox) activity in all the melanoma cell lines using the Nox Activity Assay Kit. As shown in Figure 1C, the Nox activity in melanoma cell lines was significantly greater (*P*<0.05 to *P*<0.01) than in NHM.

### Association of Nox1 expression and activity with melanoma cell migration

To examine whether over expression of Nox1 in melanoma cells correlates with migratory potential of melanoma cells, cell migration was analyzed using the Boyden chamber assay. An equal number of melanoma cells and NHM (3×10^4^) were incubated in Boyden chambers for 24 h at 37°C. After 24 h, cell migration was detected using microscope to collect photomicrographs of the cells. In general, the melanoma cell lines that have higher Nox1 activity showed a higher number of migratory cells compared to NHM. Importantly, it appears that our observation of increased migration potential was not directly associated with Nox1 protein expression but Nox activity (Figure 1D). Further, the Mel1011 cell line is deficient in β-catenin (Figure 1D, lane 8), and β-catenin has been shown to play a critical role in melanoma cell migration. Therefore, while the Mel1011 cells exhibit higher Nox activity, cell migration is impaired compared to other melanoma cell lines. A summary of our analysis of melanoma cell migration/image is presented in Figure 1E.

### Honokiol inhibits migration capacity of melanoma cells

**Figure 2:**
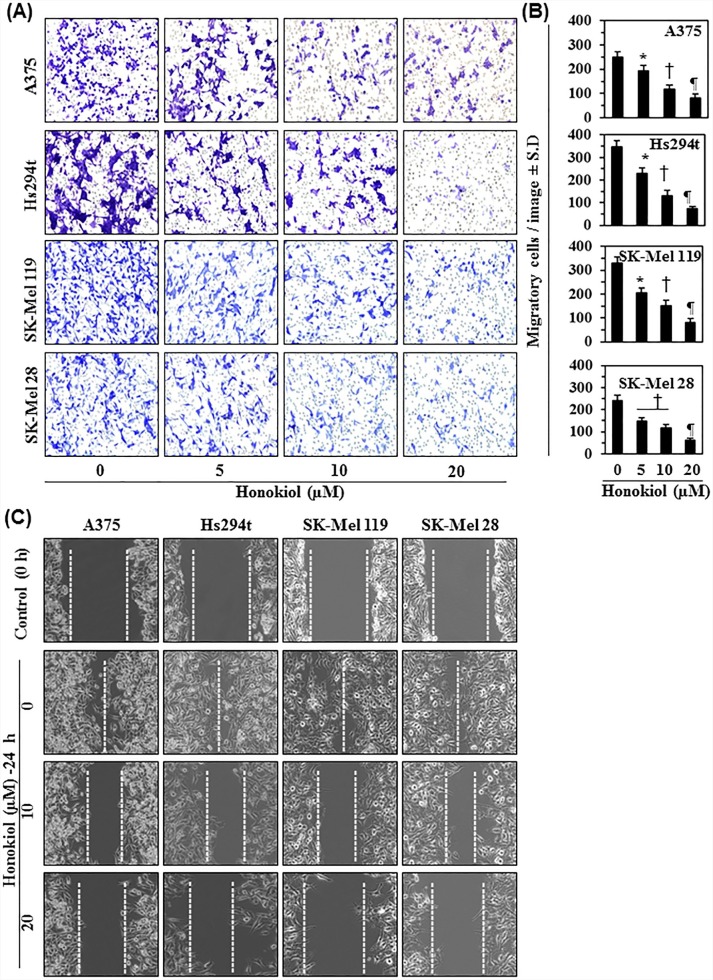
Effect of honokiol on migratory potential of melanoma cells **A.** Melanoma cell lines were treated with various concentrations of honokiol (0, 5, 10 and 20 μM) for 24 h, and cell migration was determined using Boyden chambers, as detailed in Materials and Methods. Migratory cells were detected after staining the migratory cells with crystal violet dye. Representative photomicrographs show the migratory cells in purple or blue. **B.** The migratory cells per microscopic field were counted and the results expressed as the mean number of migratory cells ± SD per image (*n* = 3). Significant difference *versus* non-honokiol-treated control group, *P<0.05, ^†^*P*<0.01, and ^¶^*P*<0.001. **C.** A scratch or wound healing assay was performed to further assess the effect of honokiol on migration capacity of melanoma cells, as detailed in Materials and Methods. The space between the broken lines delineates the gap without the presence of cancer cells.

We examined the effect of honokiol on migratory potential of different melanoma cancer cell lines. For this purpose, we selected four melanoma cell lines, A375, Hs294t, SK-Mel119 and SK-Mel28. These cell lines were treated with various concentrations of honokiol (0, 5, 10, and 20 μM) for 24 h and cell migration was determined using the Boyden chamber assay. As shown in Figure 2A, relative to honokiol-untreated control cells, treatment with honokiol reduced the migration potential of all four melanoma cell lines in a concentration-dependent manner. Migrating cells in each membrane were counted under microscope in 3-5 different fields and the resultant number of migrating cells for each cell line is summarized in terms of mean numbers of migrating cells ± SD per image (Figure 2B). The cell migration was inhibited by 20-55% (*P*<0.05-0.001) in A375 cells, 20-70% (*P*<0.05-0.001) in Hs294t cells, 40-70% in SK-Mel119 cells, and 40-80% (*P*<0.01-0.001) in SK-Mel28 cells. Inhibition occurred in a concentration-dependent manner after treatment with honokiol for 24 h.

To further verify the inhibitory effect of honokiol on melanoma cancer cell migration, a wound healing assay or scratch assay was performed, as described in the Material and methods. The major gap or wounding space between cell layers after making a wound or scratch was occupied after 24 h by the migrating melanoma cells in the control groups when cells were not treated with honokiol. Relative to non-honokiol-treated control cells, treatment with honokiol (0, 10 and 20 μM) for 24 h inhibited the migration capacity of A375, Hs294t, SK-Mel119, and SK-Mel28 cells in a concentration-dependent manner (Figure 2C). The empty space between cell layers was largely not occupied by cells treated with honokiol, and this effect was dose-dependent. The gap between the cell layers is highlighted by broken lines.

### Honokiol treatment reduces Nox1 expression and reduces intracellular ROS level in melanoma cells

**Figure 3:**
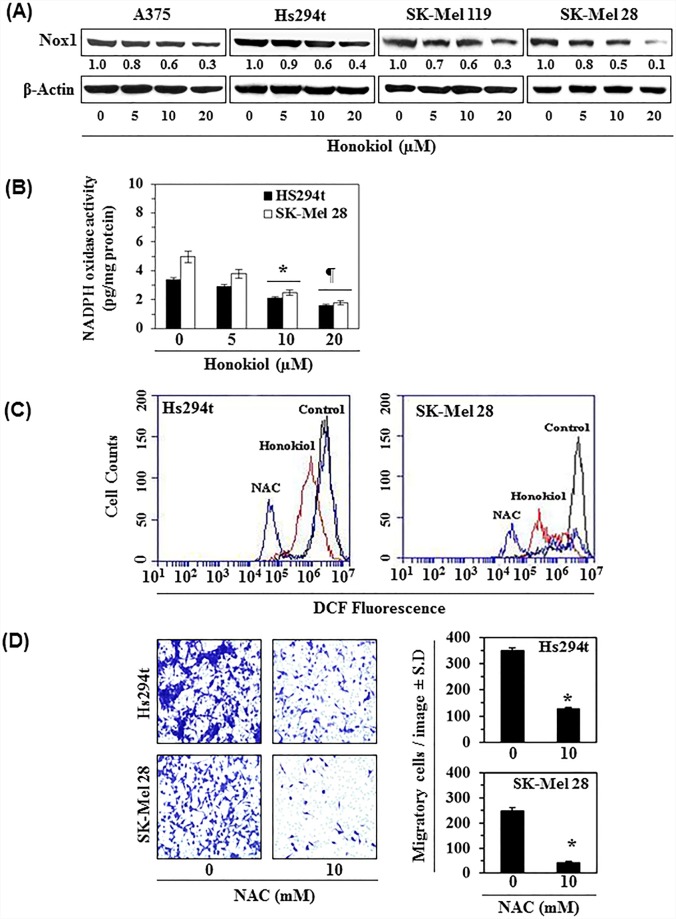
**A.** Honokiol treatment decreases the expression of Nox1 protein in melanoma cell lines. Melanoma cell lines (A375, Hs294t, SK-Mel119 and SK-Mel28) were treated with various concentrations of honokiol for 24 h. After 24 h of treatment, cell lysates were prepared and subjected to western blot analysis for the detection of Nox1 protein expression. **B.** Honokiol inhibits NADPH oxidase activity in melanoma cells. Hs294t and SK-Mel28 cells were treated with honokiol for 6 h. Cells were harvested and NADPH oxidase activity was determined using a colorimetric assay kit, as detailed in Materials and Methods. Significant inhibition versus control, *P<0.05, ^¶^*P*<0.01. **C.** Honokiol and NAC reduce the levels of oxidative stress (ROS) in melanoma cells. Hs294t and SK-Mel28 cells were treated with honokiol or NAC for 6 h and thereafter ROS production in melanoma cells was measured using flow cytometry, as described in Materials and Methods. Representative FACS histograms are shown indicating the levels of ROS production. NAC, an anti-oxidant, was used as a positive control. **D.** Treatment of melanoma cells with NAC inhibits their migratory capacity. Hs294t and SK-Mel28 cells were treated with NAC (10 mM) for 24 h and cell migration was determined using the Boyden chamber assay. Representative photomicrographs are shown with migratory cells in blue (left panels). Summary of migratory cells in different treatment groups is presented (right panels). Significant inhibition *versus* control, *P<0.001.

As honokiol treatment significantly reduced migration capacity of melanoma cells, we further determined the effect of honokiol on Nox1 protein expression in melanoma cells. A375, Hs294t, SK-Mel119, and SK-Mel28 cell lines were treated with honokiol (0, 5, 10, and 20 μM) for 24 h, and cell lysates were analyzed for Nox1 by western blot analysis. As shown in Figure 3A, treatment with honokiol decreased Nox1 expression in a dose-dependent manner. As the inhibitory effect of honokiol on Nox1 level in all the four cell lines was identical, we further checked the effect of honokiol on the levels of oxidative stress in only Hs294t and SK-Mel28 cell lines using flow cytometry. We also determined the effect of honokiol on NADPH oxidase activity. For this purpose, Hs294t and SK-Mel28 cells were treated with honokiol (0, 5, 10, and 20 μM) for 24 h. After 24 h treatment, NADPH oxidase activity was determined using colorimetric assay kit. It was found that treatment of melanoma cells with honokiol significantly reduced the activity of NADPH oxidase (*P*<0.01) compared to honokiol-untreated control cells (Figure 3B).

NADPH oxidase regulates the levels of reactive oxygen species (ROS) in cells, which play key roles as signaling molecules in many physiological and pathophysiological processes. ROS generation by the Nox1 member of the Nox family is necessary for the extracellular matrix (ECM)-degradation and actin-rich cellular structures known as invadopodia and that stimulates cell migration [16]. Here, we tested the effect of honokiol on ROS generation in melanoma cells (Figure 3C). For this purpose, Hs294t and SK-Mel28 cells were treated with honokiol for 6 h, and the intracellular level of ROS was measured by DCFH-DA assay, as described in Material and methods. As shown in Figure 3C, treatment of melanoma cells with honokiol reduced the levels of ROS generation. A similar effect was also found when melanoma cells were treated with NAC, a well-known antioxidant and scavenger of reactive oxygen species. NAC was used as positive control. To verify whether the inhibitory effect of honokiol on cell migration is mediated through its inhibitory effect on Nox1-mediated ROS generation, Hs294t and SK-Mel28 cells were treated with NAC (10 mM) for 24 h and cell migration was determined. As shown in Figure 3D (panels left and right), treatment with NAC resulted in significant inhibition of Hs294t (>60%, *P*<0.001) and SK-Mel28 (>80%, *P*<0.001) cell migration.

### Effect of diphenyleneiodonium chloride (DPI), an inhibitor of Nox1, on melanoma cell migration

**Figure 4:**
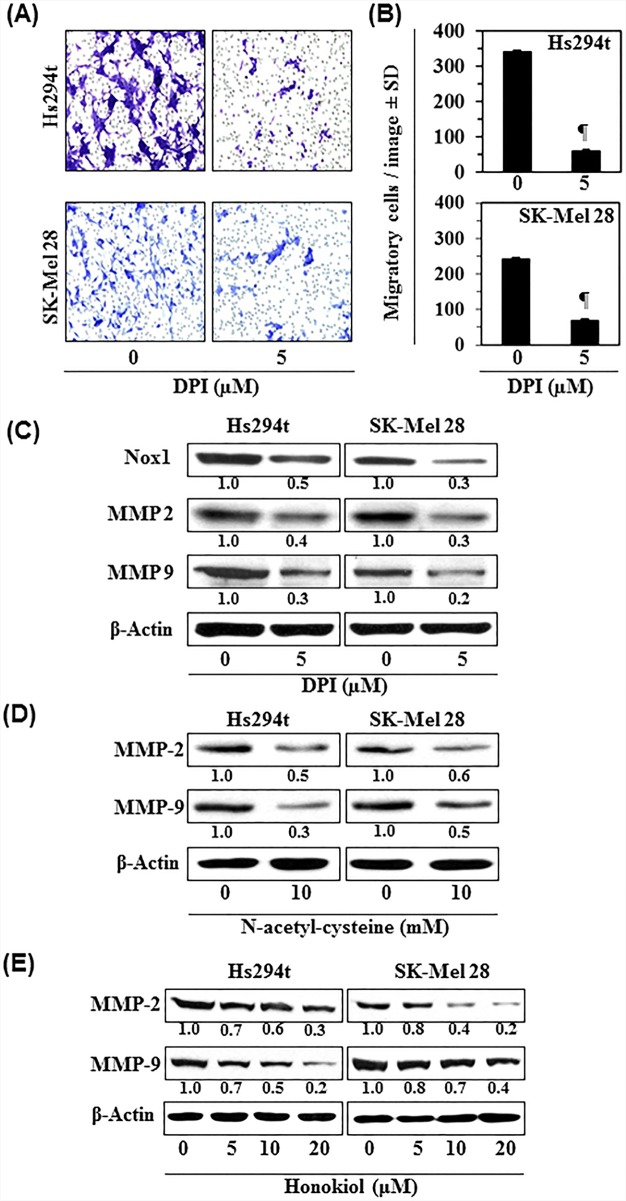
Diphenyleneiodonium chloride (DPI), an inhibitor of Nox1, inhibits melanoma cell migration **A.** Hs294t and SK-Mel28 cells were treated with DPI (5 μM) for 24 h, and cell migration was determined. Representative photomicrographs are shown from two independent experiments. **B.** The migratory blue/purple-stained cells were counted at 3-4 different places of the membrane with a microscope and the results are expressed as the mean number of migratory cells ± SD. Significant inhibition versus control group, ^¶^*P*<0.001. **C.** Inhibitory effect of DPI on the expression levels of Nox1 and MMPs in melanoma cells. After treatment of cells with DPI for 24 h, cells lysates were prepared and subjected to the analysis of Nox1, MMP-2 and MMP-9 using western blot analysis. **D & E.** Inhibitory effect of honokiol and NAC on the expression levels of MMP-2 and MMP-9 in Hs294t and SK-Mel28 melanoma cells. Cells were treated with either honokiol or NAC for 24 h, and cell lysates were subjected to the western blot analysis of MMPs.

To further verify the role of Nox1 in melanoma cell migration, Hs294t and SK-Mel28 cells were treated with DPI (5 μM), an inhibitor of Nox1, for 24 h. After 24 h treatment, cell migration was determined using Boyden chamber assay. We have found that treatment of cells with DPI significantly decreased the migration capacity of melanoma cells, as shown in representative photomicrographs (Figure 4A). Numbers of migratory cells were counted by microscopy in each group, and the results are summarized in Figure 4B. DPI treatment decreased the migration of Hs294t (85%, *P*<0.001) and SK-Mel28 (75%, *P*<0.001) cells. Western blot analysis revealed that treatment of Hs294t and SK-Mel28 cells with DPI decreased the levels of Nox1, MMP-2 and MMP-9 compared to control cells (Figure 4C).

### Effect of honokiol and NAC on matrix metalloproteinases in melanoma cells

MMPs, specifically MMP-2 and MMP-9, are well characterized for their key roles in tumor progression, cell migration and invasion of cancers including melanoma [17]. Therefore, in addition to DPI, we determined the effect of honokiol and NAC on the levels of MMP-2 and MMP-9 in melanoma cells. Hs294t and SK-Mel28 cells were treated separately with honokiol and NAC for 24 h, then cells were harvested and cell lysates were subjected to western blot analysis. The results revealed that treatment of cells with honokiol and NAC reduced expression of MMP-2 and MMP-9 in both cell lines (Figure 4D, 4E).

### Honokiol increases the accumulation of cytosolic protein p47*^phox^* while decreases the level of membrane-bound protein p22*^phox^* in melanoma cells: resultant decrease in binding of p47^phox^ and p22^phox^ proteins

**Figure 5:**
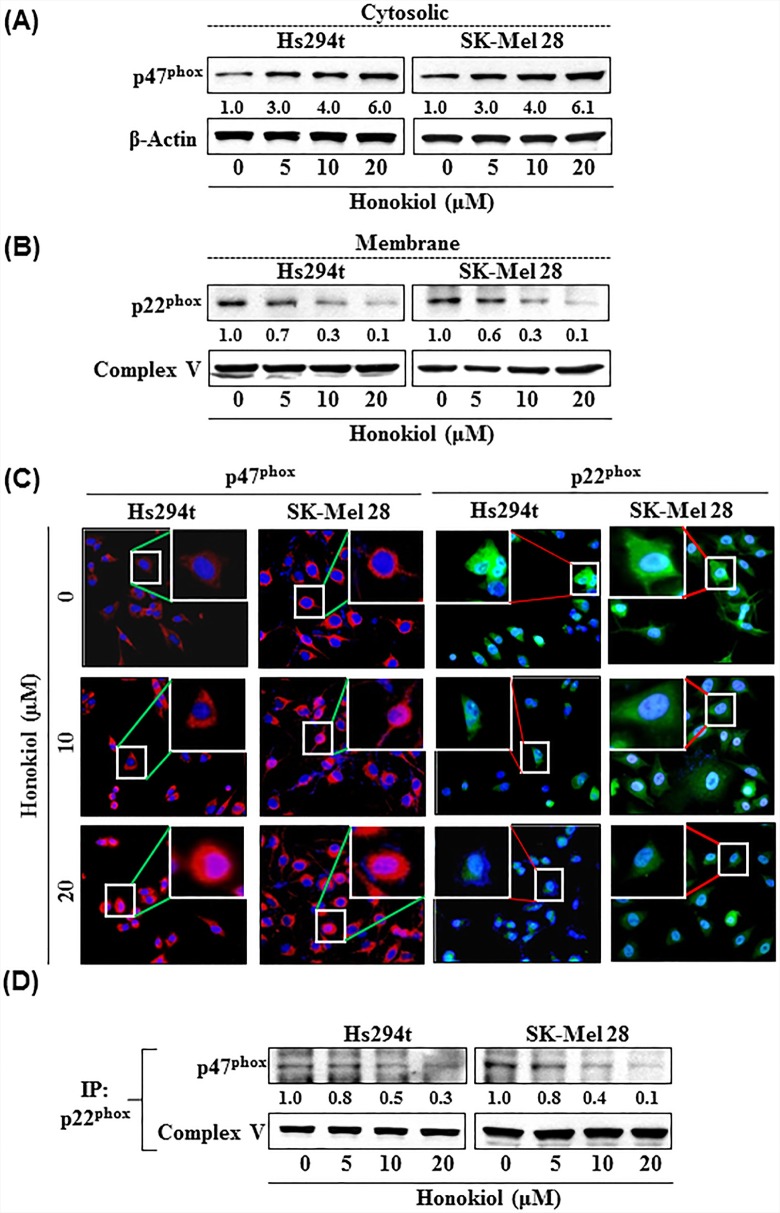
Treatment of melanoma cells (Hs294t and SK-Mel28) with honokiol increases cytosolic accumulation of p47phox protein in melanoma cells **A.** while decreasing the levels of membrane protein, p22^phox^, in a dose-dependent manner **B. C.** Immuno-cytostaining detection of cytosolic p47^phox^ and membranous protein p22^phox^ in melanoma cells after their treatment with honokiol. The cytostaining of p47^phox^ is shown in red while p22^phox^ is shown in green. For clarity, a magnified cellular staining pattern is shown as an insert. Representative photomicrographs are shown from two independent experiments. **D.** Honokiol treatment blocks the binding of p47^phox^ to p22^phox^ proteins in melanoma cells. For the binding assay, melanoma cells were treated with honokiol for 24 h, and cell lysates were prepared. The p22^phox^ protein was immunoprecipitated from cell lysates using specific antibodies. Immunoprecipitates were washed, and subjected to western blot analysis using antibody specific to p47^phox^. Complex V is a cell surface protein and is used as a loading control.

The interaction between cytosolic protein (i.e., p47*^phox^*) and membrane-bound protein (i.*e.*, p22*^phox^*) has been implicated in Nox activation [18, 19]. We therefore determined the effect of honokiol on p22*^phox^* and p47*^phox^* proteins in melanoma cells. For this purpose, Hs294t and SK-Mel28 cells were treated with honokiol for 24 h and its effect on the p22^phox^ and p47^phox^ proteins was assessed by western blot analysis. The results indicated that treatment with honokiol resulted in accumulation of cytosolic protein, p47*^phox^* (Figure 5A), and decreased levels of membrane-bound protein p22*^phox^* (Figure 5B). This effect appeared to be dose-dependent. The effect of honokiol on p47*^phox^* and p22*^phox^* protein expression in melanoma cells was further verified using cytostaining, as detailed in Materials and methods. Immuno-cytostaining detection analysis revealed that treatment of cells with honokiol resulted in increased expression levels of p47*^phox^* proteins in melanoma cells compared to non-honokiol-treated control cells (shown in red), while the staining intensity of p22*^phox^* protein, shown in green, was reduced or diminished compared to non-honokiol-treated control cells (Figure 5C). These effects of honokiol on cytosolic and membrane-bound proteins in melanoma cells may have blocked the binding of both cytosolic and membrane-bound proteins and thus may have inhibited the activation of Nox enzyme which led to the suppression of the ROS (oxidative stress) generation. We have also checked the binding levels of p47*^phox^* and p22*^phox^* proteins in melanoma cells after treatment with honokiol. The samples for generating results depicted in Figure 5D were used for this purpose. The p22*^phox^* protein was immunoprecipitated from the lysate samples from both Hs294t and SK-Mel28 cell lines and western blot analysis was performed. The results revealed that the binding of p47*^phox^* and p22^phox^ was decreased in a dose-dependent manner. These data suggest that the decreased binding of these two proteins may have contributed to reduced activation of Nox1.

### Administration of honokiol by oral gavage inhibits the extravasation capacity and establishment of tumor cell growth in internal body organs of nude mice

**Figure 6:**
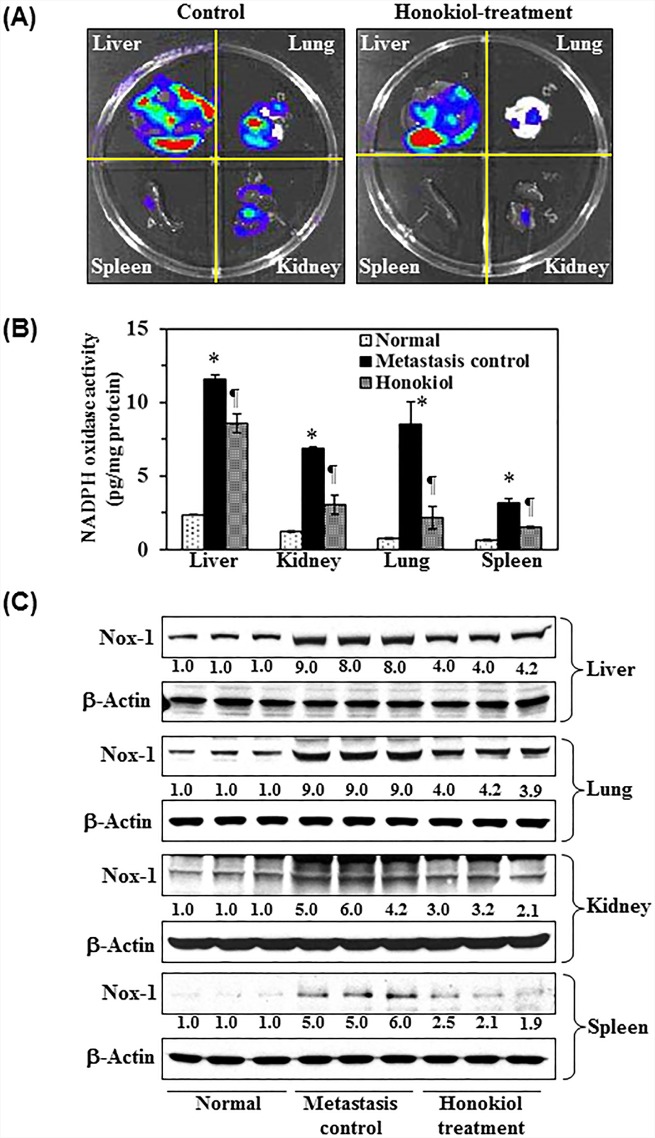
Effect of honokiol on melanoma cells invasion in vivo, NADPH oxidase activity, and the levels of Nox 1 expression in different internal body organs, such as liver, lung, spleen and kidney **A.** Mice were injected through the tail vein with A375 melanoma cells (2.5 × 10^6^/mouse) constitutively expressing luciferase and EGFP. Honokiol was administered by oral gavage, as detailed in Materials and Methods. Seven weeks after melanoma cell injection, mice were sacrificed, and internal body organs (liver, lungs, kidney and spleen) were harvested and subjected to bioluminescence imaging for the detection of melanoma cells using Xenogen IVIS200 imaging system. The area and intensity of red color indicates high accumulation or density of melanoma cells in a specific target organ, green color indicates the presence of melanoma cells but less intense than red color, blue color indicates detectable range of melanoma cells while white color indicates lack of detectable melanoma cells in that particular area. **B.** Administration of honokiol inhibits the NADPH oxidase activity in selected internal body organs of mice wherein the invasion of melanoma cells were detected (^¶^*P*<0.001, ^#^*P*<0.01) **C.** Administration of honokiol inhibits expression levels of Nox1 protein in internal body organs compared to the non-honokiol-treated but melanoma cell injected control group of mice. At the termination of the experiment, organs were harvested and lysates were prepared for the analysis of Nox1 expression level using western blot analysis.

To further verify the inhibitory effect of honokiol on melanoma cell migration, *in vivo* experiments were conducted using a nude mouse model. Melanoma cells were *i.v.* injected into the tail vein of mice that were either not treated or were orally administered honokiol. Seven weeks after injection of melanoma cells, mice were sacrificed and internal body organs, such as lungs, liver, kidney and spleens, were harvested, and subjected to image analysis, as detailed in Materials and Methods. Bioluminescence image analysis detected the presence of abundant melanoma cells in liver and lungs as shown by red color (area and intensity) while lower frequency was detected in spleen and kidney (Figure 6A). Treatment of mice with honokiol blocked migration, accumulation and growth of melanoma cells in liver and lungs compared to non-treated control animals, as reflected by the presence of red color intensity. Importantly, the metastatic melanoma cells were not detectable in lymph nodes, brain or skin by this imaging system. The levels of Nox1 activity were significantly higher (*P*<0.001) in liver, kidney and lungs compared to those detected in normal tissue samples from mice that were not injected with melanoma cells. Honokiol treatment significantly decreased (*P*<0.001) the levels of Nox1 activity in lung and liver samples compared to the non-treated (Figure 6B). We also determined the effect of honokiol on the protein expression levels of Nox1 in liver, lung, kidney and spleens tissues. Western blot analysis revealed that the expression of Nox1 protein in liver, lung, kidney and spleen was greater in mice which were injected with melanoma cells compared to the mice which were not injected with melanoma cells. Oral administration of honokiol markedly inhibited the expression levels of Nox1 in liver, lung, kidney and spleens compared to mice that did not receive honokiol (Figure 6C).

## DISCUSSION

The American Cancer Society as well as the World Health Organization reports suggest that melanoma-related deaths occur world-wide and the major cause of death is its metastatic spread beyond skin and lymph nodes [2, 20]. Current available treatment options are not sufficient to save lives, therefore, alternative innovative strategies are required to prevent or block the migratory/invasive potential of melanoma cells. The significant findings of the present study are that the treatment of human melanoma cells of different characteristics with honokiol inhibits migration capacity of melanoma cells in a dose-dependent manner. This effect is associated with the inhibition of Nox1 expression and NADPH oxidase activity in cells. Nox1 is a multi-protein complex that consists of cytosolic (p47^phox^, p40^phox^ and p67^phox^) and membrane-bound proteins (p22^phox^, gp91^phox^) that when assembled becomes activated, and initiates respiratory bursts or generation of oxidative stress [18, 21]. The generation of oxidative stress play a significant role in cancer cell progression and migration. Our data show that honokiol not only reduces the Nox1 expression or NADPH oxidase activity, but also oxidative bursts in melanoma cells. Our western blot analysis revealed that treatment with honokiol altered basal levels of p22^phox^ (membrane-bound) and p47^phox^ (cytosolic protein) in melanoma cells, which may result in blocking of interaction between p22^phox^ and p47^phox^ proteins. The p22^phox^ protein is the binding partner of p47^phox^ [22, 23], the subunits required for oxidase assembly. Failure of this interaction between membrane-bound and cytosolic proteins would lead to the inactivation of Nox in melanoma cells, and that would result in the reduction of oxidative burst, which is responsible for invasive or metastatic phenotype of melanoma cells.

The honokiol-mediated inhibition of Nox1 through its inhibitory effect on oxidative burst in melanoma cells and subsequently its anti-cell migration property is supported by the action of NAC in melanoma cells. Treatment of melanoma cells with NAC, an antioxidant, also inhibits the migration potential of melanoma cells and that is associated with the inhibition of ROS generation. The role of honokiol in inhibition of cell migration through inhibition of Nox1 expression and NADPH oxidase activity is also supported by the action of DPI in melanoma cells. Treatment of melanoma cells with DPI, an inhibitor of Nox, also blocked or reduced melanoma cell migration. Activation of MMPs, in particular MMP-2 and MMP-9, plays crucial roles in tissue matrix degradation, and thus, paves the way for cancer cell migration. To check if honokiol, NAC or DPI affect the expressions of MMPs in melanoma cells and whether it is associated with melanoma cell migration, the melanoma cells were treated with these agents. It was found that treatment of honokiol, NAC and DPI reduced the expression of MMP-2 and MMP-9 in Hs294t and SK-Mel28 melanoma cells, thus suggesting a common mechanism of action by these agents.

The inhibitory effect of honokiol on melanoma cell migration was further verified using an *in vivo* nude mouse model. A375 melanoma cells were intravenously injected through the tail vein and their migration and tumor growth in internal body organs was determined using bioluminescence imaging system. Simultaneously, we examined the inhibitory effect of honokiol, if any, on the migration potential of melanoma cells *in vivo*. Although this model system does not recapitulate the concept of tumor cell metastasis, as there was no intravasation phase of tumor cell metastasis where tumor cells migrate from the primary tumor site into the blood vascular system, this approach does help to understand how the melanoma cells pass through the extravasation phase and reach internal vital organs and grow there. Using this model and bioluminescence imaging, we detected the presence of large numbers of melanoma cells in liver and lung (Figure 6), while lower prevalence was detected in kidney. Oral administration of honokiol inhibited or blocked the migration capacity of melanoma cells as is evident by the presence of lesser numbers of melanoma cells in liver, lung and kidney compared to honokiol-untreated control mice. To verify whether this inhibitory effect on the migration of melanoma cells *in vivo* is associated with the NADPH oxidase activity and the expression of Nox protein, different organs were harvested and analyzed for these biomarkers. Administration of honokiol significantly inhibited (*P*<0.001) the NADPH oxidase activity in liver, lung and kidney compared to the control group. Additionally, the expression levels of Nox1 protein was also reduced in different organs from mice that were administered honokiol. These data explain inhibitory effects of honokiol against the migration of melanoma cells using this *in vivo* model.

In conclusion, the results from this study demonstrate that honokiol inhibits the migration capacity of melanoma cells and that this process involves: (i) the inhibitory effect of honokiol on oxidative burst/oxidative stress in melanoma cells, (ii) the suppression of NADPH oxidase activity by blocking interaction between cytosolic and membrane-bound proteins responsible for ROS generation, and that these data were supported by the bioluminescence imaging of internal body organs to detect luciferase-tagged melanoma cells. As NADPH oxidase activity has been implicated in a number of disease states, it is important to understand the exquisite regulation of this enzyme in honokiol-mediated inhibition of melanoma metastasis.

## MATERIALS AND METHODS

### Antibodies and reagents

The antibodies specific for Nox1, p47*^phox^*, p22*^phox^*, β-Actin, and secondary antibodies horseradish peroxidase-linked anti-mouse IgG, and anti-rabbit IgG were purchased from Santa Cruz Biotechnology (Santa Cruz, CA). N-acetyl-L-cysteine (NAC), 2′, 7′-dichlorofluorescein diacetate (DCFH-DA), diphenyleneiodonium chloride (DPI) and antibodies specific for MMP-2 and MMP-9 were obtained from Sigma-Aldrich Corp. (St. Louis, MO). The secondary antibodies conjugated with Alexa Fluor488 or Alexa Fluor594 were purchased from Invitrogen (Carlsband, CA). NADPH activity assay kit was purchased from AnaSpec, Inc. (Fremont, CA). Purified honokiol was purchased from Quality Phytochemicals, LLC (Edison, NJ).

### Melanoma cell lines and culture conditions

The human melanoma cells lines, A375, Hs294t, and SK-Mel 28 were purchased from the ATCC. Other melanoma cell lines, such as Mel1241, Mel1011, SK-Mel119 and Mel928 were kindly obtained from Dr. Paul Robbins (Center of Cancer Research, National Cancer Institute (Bethesda, MD). Cell lines were last authenticated in 2013, and found to be free of pathogen contamination. A brief description of cells lines is as follows: A375 and SK-Mel28 (BRAF mutated), SK-Mel119 (NRAS mutated and wild type for BRAF), Hs294t is highly metastatic but not BRAF or NRAS mutated. Mel1241 was β-catenin activated while Mel1011 was β-catenin inactivated. Majority of the cell lines are highly metastatic in nature. Most of the cell lines were cultured as monolayers in Dulbecco's modified Eagle's medium, while SK-Mel119 and SK-Mel28 were cultured in RPMI-1640 medium supplemented with 10% heat-inactivated fetal bovine serum (Hyclone, Logan, UT), 100 mg/ml penicillin and 100 mg/ml streptomycin and maintained in an incubator with 5% CO_2_ at 37°C. For the treatment of cells, honokiol was dissolved in dimethylsulfoxide (DMSO, 100 μl) and the maximum concentration of DMSO in cell culture medium was not more than 0.1% (v/v).

### Cell migration assay

The migration ability of melanoma cells was determined *in vitro* using Boyden Chambers (Gaithersburg, MD) in which the two chambers were separated with Millipore membranes (8 μM pore size), as detailed previously [15, 24]. The membranes were examined microscopically and cellular migration was determined by counting the number of stained cells on membranes in at least 3-4 randomly selected fields using an Olympus BX41 microscope with 10x magnification. Representative photomicrographs were obtained using a Qcolor5 digital camera fitted to an Olympus BX41 microscope. Cell migration experiments were repeated to verify the results.

### Wound healing or scratch assay

The wound healing or scratch assays were performed to verify the cell migration results obtained from Boyden chambers, as detailed previously [25]. Briefly, cell monolayers were wounded or scratched with a sterile 10 μl pipette tip and then washed with starvation medium to remove any detached cells from the culture plates. Cells were left either untreated or were treated with honokiol (0, 10 and 20 μM) in complete medium and kept in an incubator for 24 h. After 24 h, the medium was replaced with phosphate-buffered saline (PBS) buffer, the wound gap was visualized, and the cells were photographed using an Olympus BX41 microscope fitted with a digital camera.

### Measurement of NADPH oxidase activity

NADPH oxidase activity in normal human melanocytes and melanoma cells was measured with a colorimetric assay kit following the manufacturer's (Abcam, Cambridge, MA) protocol. The assay kit provides NADPH extraction buffer, a NADP standard, an enzyme cycling mix, and stop solution to terminate the reaction. The absorbance was measured using a microplate reader and NADPH oxidase activity was calculated following the instructions of the manufacturer and is expressed as pg/mg protein.

### Measurement of intracellular reactive oxygen species (ROS) or oxidative stress level

Intracellular ROS levels were measured in cells using flow cytometry with the redox-sensitive dye DCFH-DA [26]. The nonfluorescent DCFH-DA readily diffuses into the cells, where it is hydrolyzed to the polar derivative DCFH, which is oxidized in the presence of H_2_O_2_ to the highly fluorescent DCF. Briefly, 3×10^5^ melanoma cells (Hs294t and SK-Mel 28) were plated in 6-well culture plate and allowed to attach by overnight incubation, then treated with honokiol (20 μM) and an antioxidant N-Acetyl-L-cysteine (10 mM) for 6 h. After specific time periods, cells were harvested and stained with DCFH-DA (5μM) for 30 min at 37°C. Thereafter, cells were washed with PBS and resuspended in 1mL PBS. Fluorescence was recorded on FL-1 channel of an Accuri C6 flow cytometer (Becton Dickinson).

### Isolation of membrane and cytosolic fractions

Subcellular fractions, such as cytosolic and membrane, were prepared using the Cell Fractionation Kit (Cell Signaling Technology) following the manufacturer's instructions. The methodology is based on differential centrifugation as described by DerMardirossian et al. [27].

### Western blot analysis

Following treatment of melanoma cells with honokiol or any other agent, the cells were harvested, cell lysates prepared and western blot analysis was carried out to determine the levels of different proteins of interest, as detailed previously [25, 28]. Equal protein loading on the gel and on the membrane was verified by stripping the membrane and re-probing with an anti-β-actin antibody for cytoplasmic proteins. Complex V is a cell surface protein and anti-Complex V antibody was used as a loading control. In some cases, western blot membranes were cut into two or three parts according to the molecular weight of the proteins and subjected to incubation with different antibodies, etc. Protein ladders were used as a molecular weight marker to identify different proteins. The relative density of each band in a blot was measured using the ImageJ software (National Institute of Health). The numerical value of band density is shown under each blot, and the band density of control group was arbitrarily selected as ‘1’ and comparison was then made with densitometry values of other treatment groups.

### Immunofluorescence detection of p47*^phox^* and p22*^phox^* proteins

To determine the effect of honokiol on the formation of NADPH complex, melanoma cells (Hs294t and SK-Mel 28) were treated with various concentrations of honokiol (0, 10 and 20 μM) for 24 h. The cells were then harvested and used for cytostaining of NADPH complex proteins, such as p47*^phox^* and p22*^phox^*. Briefly, the cells were cultured on coverslips in 100 mm culture plates with or without treatment with honokiol. After 24 h of treatment, cells were fixed with chilled 4% paraformaldehyde and non-specific binding sites were blocked with 2% bovine serum albumin in PBS for 30 min. Cells were then incubated with primary antibodies specific to p47*^phox^* or p22*^phox^* for 2 h at room temperature. The cells were washed with PBS and bound antibodies were detected using an AlexaFluor-conjugated secondary antibody. Goat anti-mouse IgG labeled with green-fluorescent AlexaFluor488 dye was used for detection of p22*^phox^*, while goat anti-mouse IgG labeled with red-fluorescent AlexaFluor594 was used for the detection of the expression of p47*^phox^*. Cells were finally mounted with Vectashield mounting medium for fluorescence with DAPI (Vector Laboratories, Burlingame, CA). Immunofluorescence detection was performed using a fluorescence microscope and representative images were collected.

### Generation of EGFP/luciferase reporter A375 melanoma cells

To facilitate detection of experimental metastasis *in vivo*, A375 cells were transduced with a vesicular stomatits virus G envelope (VSV-G) pseudotyped lentiviral vector for constitutive expression of both firefly luciferase and enhanced green fluorescence protein (EGFP). The vector (designated K2947) comprised the mouse CMV promoter, followed by fire fly luciferase, the encephalomyocarditis internal ribosomal entry site (IRES), a puromycin resistance gene and the enhanced green fluorescence protein (EGFP), wherein puromycin and EGFP were fused in-frame at their 3′ and 5′ ends, respectively, with the “self-cleaving” T2A peptide-coding sequence [29] (CMV-luciferase-IRES-puro.T2A.EGFP). The lentiviral vector, the packaging construct, and the VSV-G plasmid DNAs were co-transfected into 293T human embryo kidney cells to create infectious, replication defective, lentiviral vector-containing particles as described previously [30]. A375 cells were transduced with the vector using a multiplicity of infection of approximately 5. Stable, expression-positive A375 cells were selected by supplementing the culture medium with 5 mg/ml of puromycin for 5 days.

### Detection and analysis of metastatic melanoma cells in internal body organs of athymic nude mice using bioluminescence imaging

Experimental metastasis of melanoma cells was determined by intravenous injection of A375 cells (2.5×10^6^) constitutively expressing both luciferase and green fluorescent protein (GFP) into nude mice as a model. Female athymic nude mice (4-6 weeks of age) were purchased from the National Cancer Institute (Bethesda, MD) and housed in the animal resource facility at the University of Alabama at Birmingham in accordance with the Institutional Animal Care and Use Committee (IACUC) guidelines. Briefly, mice were divided into three groups of 5 animals each. Honokiol was administered (100 mg/kg body weight) in supplementation with 0.5% carboxymethyl cellulose dissolved in 200 μl sterile water/mouse or vehicle via oral gavage 5x per week for seven weeks. After seven weeks, the mice were sacrificed and their vital organs (liver, lungs, kidneys, and spleen) were harvested. The presence of metastatic melanoma cells in these organs was detected by bioluminescence imaging after spraying D-luciferin using Xenogen IVIS200 imaging system, following the reported protocols [31, 32].

### Statistical analysis

For migration assays, the data from honokiol-, NAC- and DPI-treated groups were compared with control group separately using one-way analysis of variance (ANOVA) followed by *post hoc* Dunn's test using GraphPad Prism version 4.00 for Windows, GraphPad Software, (San Diego, CA), USA, www.graphpad.com. In each case *P*<0.05 was considered statistically significant.
